# NADPH oxidases as potential pharmacological targets against increased seizure susceptibility after systemic inflammation

**DOI:** 10.1186/s12974-018-1186-5

**Published:** 2018-05-12

**Authors:** Wan-Yu Huang, Shankung Lin, Hsuan-Ying Chen, Ya-Ping Chen, Ting-Yu Chen, Kuei-Sen Hsu, Hung-Ming Wu

**Affiliations:** 1 Institute of Basic Medical Sciences, College of Medicine, National Cheng Kung University, Tainan, Taiwan; 2 Pediatrics of Kung-Ten General Hospital, Taichung City, Taiwan; 3 Inflammation Research & Drug Development Center, Changhua Christian Hospital, Changhua, Taiwan; 4 Department of Neurology, Changhua Christian Hospital, Changhua City, Taiwan; 5 Institute of Acupuncture, School of Chinese Medicine, China Medical University, Taichung City, Taiwan

**Keywords:** NADPH oxidase, Diphenyleneiodonium, Lipopolysaccharide, Seizure, Cytokine, Neuroninflammation

## Abstract

**Background:**

Systemic inflammation associated with sepsis can induce neuronal hyperexcitability, leading to enhanced seizure predisposition and occurrence. Brain microglia are rapidly activated in response to systemic inflammation and, in this activated state, release multiple cytokines and signaling factors that amplify the inflammatory response and increase neuronal excitability. NADPH oxidase (NOX) enzymes promote microglial activation through the generation of reactive oxygen species (ROS), such as superoxide anion. We hypothesized that NOX isoforms, particularly NOX2, are potential targets for prevention of sepsis-associated seizures.

**Methods:**

To reduce NADPH oxidase 2-derived ROS production, mice with deficits of NOX regulatory subunit/NOX2 organizer p47^phox^ (p47^phox−/−^) or NOX2 major subunit gp91^phox^ (gp91^phox−/−^) were used or the NOX2-selective inhibitor diphenyleneiodonium (DPI) was used to treat wild-type (WT) mice. Systemic inflammation was induced by intraperitoneal injection of lipopolysaccharide (LPS). Seizure susceptibility was compared among mouse groups in response to intraperitoneal injection of pentylenetetrazole (PTZ). Brain tissues were assayed for proinflammatory gene and protein expression, and immunofluorescence staining was used to estimate the proportion of activated microglia.

**Results:**

Increased susceptibility to PTZ-induced seizures following sepsis was significantly attenuated in gp91^phox−/−^ and p47^phox−/−^ mice compared with WT mice. Both gp91^phox−/−^ and p47^phox−/−^ mice exhibited reduced microglia activation and lower brain induction of multiple proconvulsive cytokines, including TNFα, IL-1β, IL-6, and CCL2, compared with WT mice. Administration of DPI following LPS injection significantly attenuated the increased susceptibility to PTZ-induced seizures and reduced both microglia activation and brain proconvulsive cytokine concentrations compared with vehicle-treated controls. DPI also inhibited the upregulation of gp91^phox^ transcripts following LPS injection.

**Conclusions:**

Our results indicate that NADPH oxidases contribute to the development of increased seizure susceptibility in mice after sepsis. Pharmacologic inhibition of NOX may be a promising therapeutic approach to reducing sepsis-associated neuroinflammation, neuronal hyperexcitability, and seizures.

**Electronic supplementary material:**

The online version of this article (10.1186/s12974-018-1186-5) contains supplementary material, which is available to authorized users.

## Background

Seizure is a common acute complication of sepsis and ensuing systemic inflammation, particularly in neonates and infants [1, 2]. Seizures are caused by genetic factors or acquired clinical pathologies that disrupt the excitatory/inhibitory balance within brain circuits. During inflammation, it is believed that hyperexcitation of neurons, resulting from the release of signaling factors such as cytokines and reactive oxygen species (ROS), leads to enhanced seizure predisposition [3–5] and subsequent neuroplastic changes and neurodegeneration, which may eventually induce a chronic seizure syndrome [6, 7].

Growing evidence indicates that transient systemic inflammation can alter neuronal properties and behavior, particularly during critical periods of brain development, resulting in programmed neuron death in adulthood. For instance, a single intraperitoneal (i.p.) injection of the potent immune system activator lipopolysaccharide (LPS) administered to postnatal rats can cause increased seizure susceptibility [7] and alter NMDA receptor subunit mRNA expression in the adult brain [8]. Qin and colleagues also reported that a single systemic LPS injection to adult mice induced chronically persistent activation of microglia and progressive loss of dopaminergic neurons in the substantia nigra [6]. Although brief peripheral inflammation usually does not damage the mature brain, it can induce transient functional deficits in behavior and an acute inflammatory response in the brain similar in some respects to that in the periphery, including elevation of the same proinflammatory cytokines [9, 10]. This induced neuroinflammation can be transient or long-lasting depending on the age at insult [6, 7, 11–13]. Collectively, such studies indicate that both immature and mature brains can be permanently modified after a single brief episode of systemic inflammation, resulting in increased neuronal excitability and behavior changes.

Microglia, the resident macrophage-like immune cells of the central nervous system (CNS), sense local pathological changes, induce and amplify the inflammatory response, and exert additional neuromodulatory effects through dynamic regulation of astroglial activity [14]. In response to systemic inflammation, microglia become activated and may then damage neurons through excessive production and release of neurotoxic factors, including proinflammatory cytokines and ROS. Both clinical reports and experimental studies in vivo and in vitro suggest that these cytokines may increase neuronal excitability and seizure susceptibility [15]. Riazi and colleagues reported that microglia-derived TNFα contributes to neuronal hyperexcitability and exacerbates pentylenetetrazole (PTZ)-induced seizures following peripheral inflammation [4]. Conversely, inhibition of action of the cytokine interleukin (IL)-1β in the brain by application of an exogenous IL-1 receptor antagonist (IL-1ra) or by enhancing endogenous IL-1ra expression can suppress seizure induction or reduce seizure severity in animal models [16–18]. In addition to tumor necrosis factor alpha (TNFα) and IL-1, cytokines reported to modulate neuronal excitability and seizure susceptibility include IL-6 [19, 20], monocyte chemoattractant protein-1 (MCP-1/CCL2) [5], IL-10 [21], and IL-12 [21].

NADPH oxidase 2 (NOX2), also called phagocytic NADPH oxidase (PHOX), is a member of the NADPH oxidase (NOX) family of enzymes implicated in phagocytic bactericidal and fungicidal activities through ROS production. NOX enzymes, principally NOX2, are also involved in microglia activation. When microglial NOX2 is activated, several ROS including superoxide anion (O_2_^**·**−^), hydroxyl radical (OH^**·**−^), lipid hydroperoxides, and byproducts (e.g., H_2_O_2_), are generated that can directly damage neurons through extracellular pathways as well as induce further ROS generation and the expression of proinflammatory genes such as IL-1β, TNFα, and inducible nitric oxide synthase (iNOS) [22, 23].

We hypothesized that phagocytic NADPH oxidase (NOX2) within the brain, mainly expressed in microglia, plays an essential role in increased neuronal excitability following systemic inflammation. While targeting inflammation has long been proposed as a potential therapeutic strategy for prevention of neuroinflammation-associated seizures [18, 24–26], there are few studies showing that this is an effective approach. Therefore, the main purpose of this study was to investigate whether NOX2 plays a critical role in seizure susceptibility after systemic inflammation and whether post-treatment with the widely used and long-acting NOX2 inhibitor, diphenyleneiodonium (DPI) [27], can attenuate the enhanced seizure susceptibility induced by systemic inflammation. By comparing seizure susceptibility among mice genetically deficient in NOX2 complex subunits gp91^phox^ and p47^phox^, DPI-treated mice, and wild types (WT), we demonstrate that NOX2-derived ROS contribute to neuronal hyperexcitability and the increased seizure susceptibility following LPS-induced inflammation. Thus, pharmacological NOX2 blockade is a potential therapeutic strategy for prevention of sepsis-associated seizures.

## Methods

### Animals and lipopolysaccharide-induced sepsis model

Male C57BL/6J mice (8–9 weeks old, 25–30 g) were purchased from the National Lab Animal Center (Taiwan). The NOX2 subunit knockout mice B6.129S6-Cybbtm1Din/J mice (gp91^phox−/−^, JAX stock 002365) and B6(Cg)-Ncf1<m1J>/J mice (p47^phox−/−^, JAX Stock 004742) were obtained from The Jackson Laboratory (Bar Harbor, ME, USA). The PHOX^−/−^ mutation is maintained on a C57BL/6J background; therefore, C57BL/6J (PHOX^+/+^) mice were used as control animals for this study. The animals were housed in a specific pathogen-free room at 21 °C under a 12-h/12-h artificial light/dark cycle with free access to feed. Experiments were performed using age- and weight-matched male animals. All procedures were approved by the Animal Care and Use Committee of Changhua Christian Hospital. The mouse model of sepsis was induced by intraperitoneal (i.p.) injection of 4 mg/kg lipopolysaccharide (LPS), modified from a previous study in mice [28]. In total, 352 of mice were used in this study, including 248 of WT mice, 52 of p47^phox−/−^ mice, and 52 of gp91^phox−/−^ mice.

### Diphenyleneiodonium treatment

Diphenyleneiodonium (DPI) (Sigma-Aldrich, St. Louis, MO, USA) is a pharmacological inhibitor of NADPH oxidase. In the present study, we assessed the efficacy of DPI against PTZ-induced seizure susceptibility in LPS-treated mice. Diphenyleneiodonium was injected i.p. at a dose of 0.01, 0.1, or 1 mg/kg at indicated time points after LPS injection.

#### Determination of seizure susceptibility to pentylenetetrazol

To assess seizure susceptibility, mice were injected i.p. with 60 mg/kg pentylenetetrazole (PTZ) 48 h after LPS or vehicle treatment (Sigma, St. Louis, MO, USA). Pentylenetetrazole may have multiple mechanisms of action in neurons, one of which is strongly correlated with its affinity for the picrotoxin binding site on the GABA_A_ receptor complex [29, 30]. Seizure activity was video recorded during an observation period of 2 h after PTZ injection. Behavioral seizures were scored every 5 min according to a previously defined scale [31, 32] as follows: stage 1 (exploring, sniffing, and grooming ceased and immobility); stage 2 (forelimb and/or tail extension, appearance of a rigid posture); stage 3 (isolated myoclonic jerks, with brief twitching movements); stage 4 (forelimb clonus and partial rearing); stage 5 (forelimb clonus, rearing, and falling); and stage 6 (generalized tonic-clonic activity with loss of postural tone, often resulting in death).

### Plasma levels of TNFα after peritoneal LPS administration

To assess the acute effects of LPS on systemic inflammation, blood samples were obtained from the cheek of the wild-type and NOX2 knockout mice 1 h after LPS injection (4 mg/kg, i.p.). The plasma samples were stored at − 80°C until they were assayed for TNFα concentration according to the manufacturer’s instructions (Duo set kit; R&D Systems, Minneapolis, MN, USA).

### Microglia-enriched cultures

Primary enriched-microglial cultures were prepared from WT, gp91^phox−/−^, and p47^phox−/−^ pups using a previously described protocol [28, 33]. Briefly, the whole brain obtained from a 0–1-day-old mouse pup was carefully removed, and the olfactory bulbs, cerebellum, brain stem, meninges, and blood vessels were separated. The remaining brain tissue was triturated into a single-cell suspension, which was then centrifuged at 500×*g* for 6 min at 4 °C. The pellet was resuspended in the warm mixed glial culture maintenance medium. The cells were cultured with a seeding density of five brains per 175-cm^2^ flask. The culture medium was refreshed every 3 days. Approximately 14–16 days later, microglia were shaken out at 180 rpm for 30 min to 1 h at 37 °C. After centrifugation, the resuspended microglia were seeded in a 24-well plate at a density of 7.5 × 10^4^/well. After storage overnight, cells were treated with LPS (5 ng/ml), TNFα (500 pg/ml), or IL-1β (500 pg/ml). The purity of microglia was > 98%, confirmed by staining with F4/80 antibody.

### Western blotting analysis

For protein lysate extraction, dissected mouse brain tissue (e.g., hippocampus) was homogenized and lysed in ice-cold modified radioimmunoprecipitation assay (RIPA) buffer containing 50 mM Tris-HCl (pH 7.4), 1% Nonidet P-40, 150 mM NaCl, 1 mM EDTA, 1 mM phenylmethylsulfonyl fluoride, 10 μg/ml each of aprotinin, leupeptin, and pepstatin, 1 mM Na_3_VO_4_, and 1 mM NaF. Immunobloting analysis was performed as described [28, 33]. Briefly, total proteins from each sample were fractionated by SDS-PAGE and then transferred to polyvinylidene difluoride (PVDF) membranes. Membranes were probed with antibodies against the proteins including gp91 (Cat# sc-5827) and β-actin(Cat# sc-47,778) (the gen loading control) from Santa Cruz Biotechnology (Dallas, TX), Cruz, iba-1(Cat# GTX100042), GFAP(Cat# GTX85454) and BDNF(GTX62495) from GeneTex Biotechnology (Irvine, CA), and hsp 90 (Cat# 4877) (the gel loading control) from Cell Signaling Technology (Danvers, MA) at 1/1000 dilution. Immunoblotted membranes were then washed for 10 min in 0.1% Tris-buffered saline-Tween 20 (TBST), incubated in horseradish peroxidase (HRP)-conjugated secondary antibody (1/10,000 dilution) for 1 h, and washed again in TBST. Signals were visualized and quantified using the GeneGnome chemiluminescence imaging system (Syngene, Maryland).

### Measurement of cytokines in brain supernatant

Total brain proteins were extracted using a protocol modified from previous studies on cytokine/chemokine panels [10, 34]. Briefly, temporal lobe tissue was weighed and homogenized with a bench top homogenizer (Kinematica, Switzerland) in a 5× volume of extraction buffer containing 20 mM Tris HCl, 0.15 M NaCl, 2 mM EDTA, 1 mM EGTA, and Protease Inhibitor Cocktail (Sigma, St. Louis, MO). Samples were centrifuged at 1000×*g* for 10 min at 4 °C, and the supernatants were centrifuged again at 20,000×*g* for 40 min at 4 °C to remove any remaining debris. Protein concentration was measured using a BCA Protein Assay Kit (Pierce Biotechnology, Rockford, IL). All cytokine and chemokine measurements were performed using MERCK Millipore Presents MILLIPLEX® MAP multiplex Assays (St. Charles, MO, USA) by the procedures reported previously [10, 34]. Concentrations of TNFα, IL-1β, IL-6, CCL2, IL-10, and IL-12 were simultaneously determined in brain samples using a LINCOplexTM mouse cytokine kit and a Luminex 200 reader (Luminex Corp., Austin, TX, USA). Concentrations were calculated by generating a calibration curve using recombinant cytokines diluted in brain sample extraction buffer and StatLIAs software (Brendan Scientific Corp., Carlsbad, CA, USA). Cytokine and chemokine concentrations were then normalized to total protein for each sample.

### Real-time RT-PCR analysis

To assess the expression of proinflammatory factors TNFα, IL-1β, IL-6, CCL2, IL-10, and IL-12, and gp91 in response to LPS-induced systemic inflammation, mouse brains were prepared at the indicated time points following LPS treatment. Total RNA was prepared from temporal lobe tissues using the RNeasy micro kit (Qiagen, Valencia, CA, USA) and reverse transcribed into cDNA using the Super Script-III First-strand Synthesis System kit (Invitrogen, Carlsbad, CA, USA). Real-time PCR was performed using the Rotor-Gene Q cycler (Qiagen, Valencia, CA, USA) according to the manufacturer’s protocol. All experiments were conducted in triplicate using TaqMan Gene Expression Master mix (Applied Biosystems) and TaqMan Gene Expression Assays, with optimized primer and TaqMan MGB probe sets (Applied Biosystems, Foster City, CA, USA). The PCR primer pairs and TaqMan MGB probes (assay-IDs) (Applied Biosystems) were as follows: Mm00443260_gl (TNFα), Mm00434228_ml (IL-1β), Mm00446190_ml (IL-6), Mm00441242_ml (CCL2), Mm01288386_ml (IL-10), Mm00434174_ml (IL-12b), and Mm00607939Msl (β-actin). A total of 40 cycles of PCR was performed as follows: activation of AmpliTaq Gold Enzyme (10 min at 95°C), denaturation (15 s at 95 °C), and annealing/extension (1 min at 60 °C). Relative expression levels were calculated using the comparative threshold cycle (Ct value) method and normalized to the ΔCt of *β-actin*.

### Immunofluorescence staining

To assess the effects of LPS-induced systemic inflammation on CNS glia, mouse brain sections were prepared for immunostaining. Mice were deeply anesthetized and perfused with 4% paraformaldehyde (PFA) 24 h after vehicle or LPS treatment. The brain was dissected and post-fixed overnight in 4% PFA at 4 °C. Coronal frozen sections (30 μm thick) through hippocampi were prepared as described [28]. Brain sections were permeabilized with 1% Triton X-100 in PBS for 10 min and then blocked in 1% bovine serum albumin in PBST for 1 h. To identify microglia, slices were incubated overnight at 4 °C in blocking buffer containing an antibody against ionized calcium binding adaptor molecule 1 protein (iba-1) (Cat# GTX100042) from GeneTex Biotechnology (Irvine, CA), at 1/200 dilution. The brain sections were then also incubated with donkey anti-rabbit-FITC antibody (1200) at room temperature for 1 h. Sections were then counterstained with 4′, 6-diamidino-2-phenylindole (DAPI) for 5 min at room temperature and observed by fluorescence microscopy. Microglial activation was determined by morphology changes from round and small (resting state) to rod- and/or amoeboid shaped with a significant enlargement of cell size (activated state). We used a stereological approach to count the number of resting and activated microglia from three sections of the hippocampus per mouse (*n* = 3 mice/group), including dentate gyrus (DG) and CA3 subregions on a ZEISS-Axio Observer Z1, HAL 100 microscope (ZEISS, Germany) under bright-field optics [33, 35]. Briefly, images were taken from DG and CA3 subregions at − 2.18 to − 2.54 mm from the bregma. Coordinates for the DG were taken centered from 1.4 to 1.7 mm medial and 1.7 to 1.9 mm ventral. Coordinates for the CA3 were from 2.1 to 2.4 mm medial and from 1.8 to 2.1 mm ventral. Activated microglia were distinguished from resting microglia by the presence of shorter, less-ramified processes and rod-and/or amoeboid appearance. Quantification of microglia was done by a single experimenter who was blind to each animal’s treatment, by counting the total number of iba-1 positive cells at 200× magnification in a rectangular region of interest (300 × 400 μm^2^). Results are presented as means ± standard error of the mean (SEM) in the counted area.

### Statistical analysis

Values are presented as mean ± SEM. Paired group means were compared by Student’s *t* test. The efficacy of *NOX2* deletion or DPI treatment against PTZ-induced seizure severity was assessed by two-way repeated measures ANOVA, adjusted by Bonferroni tests. Multiple group means were compared by one-way ANOVA, followed by Bonferroni post hoc tests. All analyses were conducted using the software GraphPad Prism 7 (La Jolla, CA). Differences were considered significant at *p* < 0.05.

## Results

### NOX2 deletion reduces the increased seizure susceptibility to pentylenetetrazole following LPS-induced systemic inflammation

**Fig. 1:**
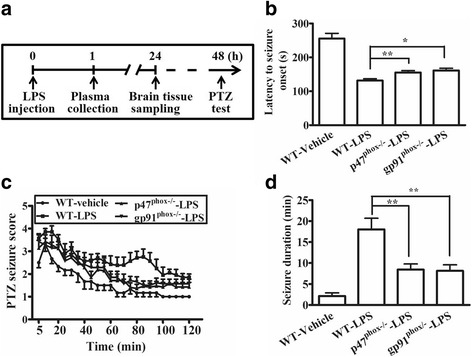
NADPH oxidase deletion reduced the increased pentylenetetrazole (PTZ)-induced seizure susceptibility following lipopolysaccharide (LPS) stimulation. **a** The experiment protocol is schematized. Wild-type (WT), gp91^phox−/−^, and p47^phox−/−^ mice were injected intraperitoneally (i.p.) with 4 mg/kg lipopolysaccharide (LPS). Plasma samples were collected 1 h later for TNFα quantification and some mice were sacrificed 24 h later for the preparation of brain sections and transcript assays. Two days later, seizure susceptibility to 60 mg/kg PTZ (i.p.) was evaluated (*n* = 6 or 7 mice per genotype). **b** The latency to initial seizure onset (clonic with/without tonic convulsion) after PTZ administration. Data are presented as mean ± SEM. Bonferroni post hoc test vs. LPS-treated WT group; *p < 0.05, **p < 0.01. **c** Seizure susceptibility of gp91^phox−/−^, and p47^phox−/−^ and WT mice scored once every 5 min over the 2-h period following PTZ injection. Two-way repeated measures ANOVA revealed that the main effect for LPS-treated WT, gp91^phox−/−^, and p47^phox−/−^groups yielded an F ratio of F(2, 432) = 34.12, *p* < 0.0001, Bonferroni post-test analysis further revealed significant difference between the WT group and gp91^phox−/−^ (F(1,288) = 42.63; *p* < 0.0001) and between WT and p47^phox−/−^ (F(1,288) = 54.86; *p* < 0.0001), but no difference between p47^phox−/−^ and gp91^phox−/−^ groups ((F(1,288) = 1.16; *p* = 0.282). **d** The total duration (min) of seizure behavior ≥ stage 4. Data are presented as mean ± SEM. Bonferroni post hoc test vs. vehicle-treated or LPS-treated WT group; **p < 0.01, ***p < 0.001

To elucidate the role of NOX2 in seizure incidence following systemic inflammation, we induced systemic inflammation in adult (8–9-week-old) wild-type, gp91^phox−/−^, and p47^phox−/−^ mice with a single dose of LPS via i.p. injection and subsequently determined PTZ-induced (60 mg/kg, i.p.) seizure susceptibility by scoring the severity and duration of the PTZ-induced seizure every 5 min for 2 h (Fig. 1a). The latency to initial seizure onset (clonic with/without tonic convulsion) after PTZ administration was significantly decreased in LPS-treated gp91^phox−/−^ and p47^phox−/−^ groups, compared with the LPS-treated WT group (Fig. 1b). In addition, the seizure susceptibility to PTZ of all three genotype groups treated with LPS was significantly increased, compared with the vehicle-treated (saline) control group (all *p* values < 0.0001 by two-way repeated-measures ANOVA). The main effect for LPS-treated WT, gp91^phox−/−^, and p47^phox−/−^ groups yielded an F ratio of F(2,432) = 34.12, *p* < 0.0001, indicating a significant difference between these three groups in susceptibility to PTZ-induced seizure. The Bonferroni test further revealed a significant difference between the WT group and gp91^phox−/−^ (F(1,288) = 42.63; *p* < 0.0001) and between WT and p47^phox−/−^ (F(1,288) = 54.86; *p* < 0.0001), but no difference between p47^phox−/−^ and gp91^phox−/−^ groups ((F(1,288) = 1.16; *p* = 0.282). The results indicate that both LPS-treated gp91^phox−/−^ and p47^phox^ groups exhibited significantly attenuated the increased seizure susceptibility, compared with the LPS-treated WT group (Fig. 1c). We measured the duration of stage 4–6 seizures among the mice receiving LPS or vehicle control. The results showed that the seizure duration was 2.14 ± 0.73 (mean ± SEM), 18.00 ± 2.67, 8.14 ± 1.44, and 8.43 ± 1.36 mins, for the vehicle-treated WT, LPS-treated WT, LPS-treated gp91^phox−/−^, and LPS-treated p47^phox−/−^ groups, respectively (Fig. 1d). Notably, there was no difference between gp91^phox−/−^ and p47^phox−/−^ mice following LPS injection (*p* > 0.05). These results strongly suggested that NOX2 was a critical component of the elevation in seizure susceptibility following systemic inflammation.

### NOX2 deletion attenuated microglial activation and the upregulation of proconvulsive cytokines following LPS injection

**Fig. 2:**
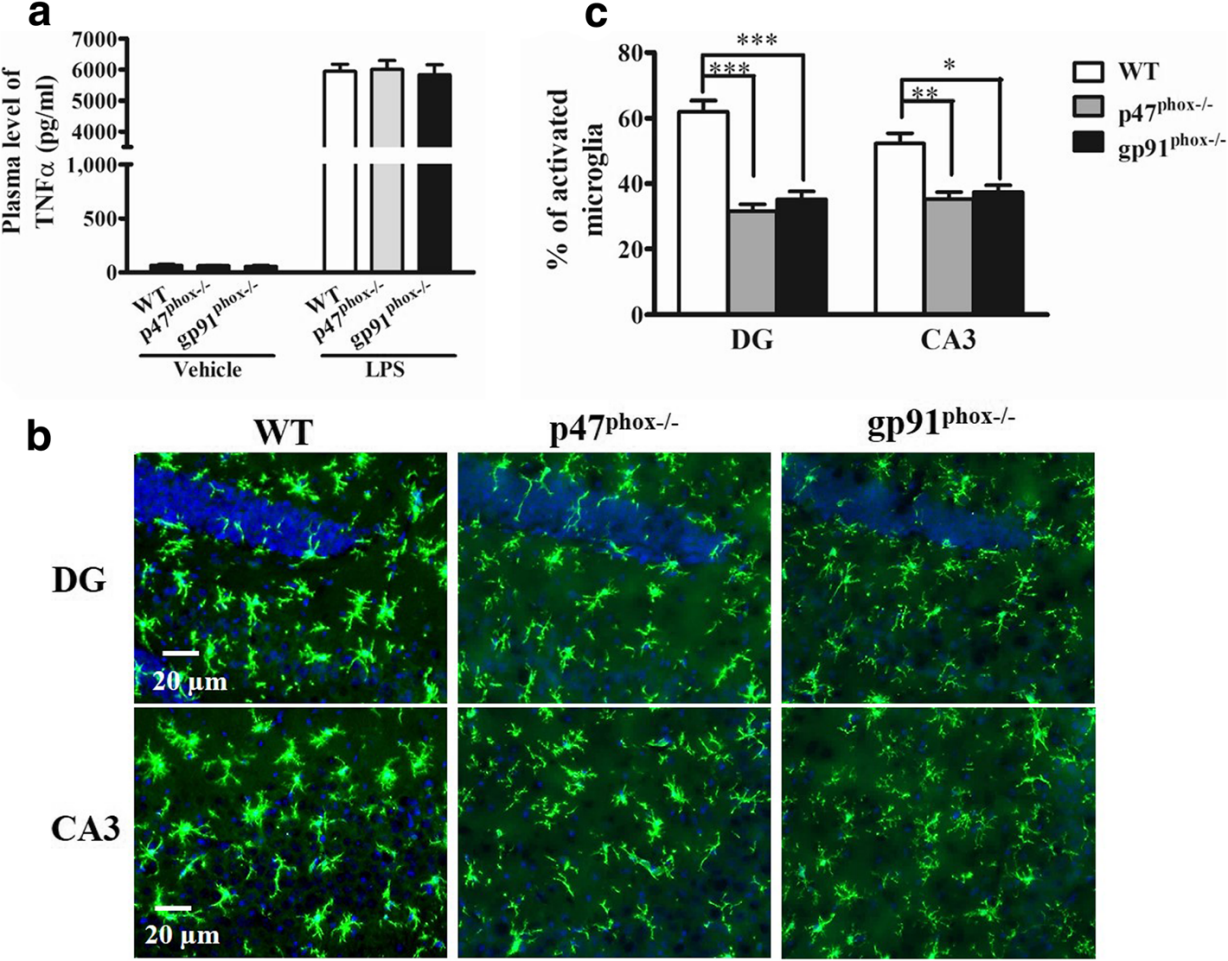
Effects of NADPH oxidase subunit deletion on plasma TNFα levels and microglial activation after LPS injection. **a** The plasma TNFα levels were not affected by NOX2 subunit knockout (gp91^phox−/−^ and P47^phox−/−^) 1 h after LPS. Data are presented as mean ± SEM; *n* = 5/group. Bonferroni post hoc test vs. vehicle-treated or LPS-treated wild-type (WT) group; *p* > 0.05. **b** WT, gp91^phox−/−^, and P47^phox−/−^ mice were sacrificed 24 h after 4 mg/kg LPS injection (i.p.). Brain sections were immunostained for the microglial marker Iba-1. Representative images of the stained microglia in the dentate gyrus (DG) and CA3 regions of mouse hippocampus (*n* = 3 mice per genotype) are shown. **c** Activated microglia were identified by increased cell size and irregular shape. The proportions of activated microglia in hippocampus were estimated. Data are mean ± SEM of values from three animals per genotype. Bonferroni post hoc test vs. LPS-treated WT mouse group; *p < 0.05, **p < 0.01, ***p < 0.001

**Fig. 3:**
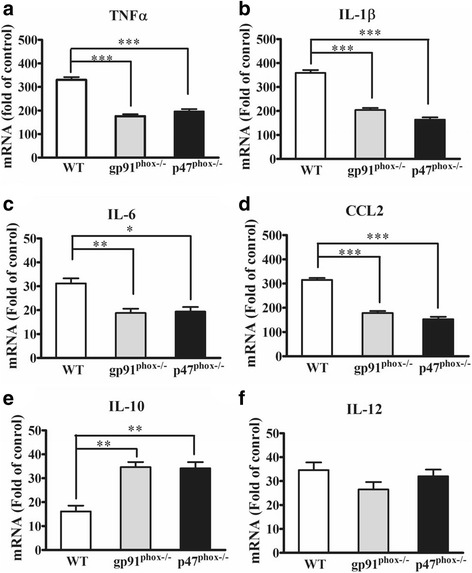
NADPH oxidase deletion attenuated the upregulation of proconvulsive cytokines in mouse brain following LPS stimulation. Wild-type (WT), gp91^phox−/−^ and P47^phox−/−^ mice were treated with saline (vehicle) or 4 mg/kg LPS (i.p.). Mouse brains (*n* = 5 per genotype) were harvested 1.5 h after LPS injection. The mRNA levels of TNFα, IL-1β, CCL2, IL-6, IL-10, and IL-12 was determined by RT-qPCR (**a**-**f**). Cytokine mRNA expression was normalized to β-actin mRNA expression (internal control). Fold change was calculated by comparing the expression of each cytokine of the LPS-treated mice to that of the genotype-matched saline-treated mice. Data present the mean ± SEM. Bonferroni post hoc test vs. corresponding WT control; *p < 0.05, **p < 0.01, ***p < 0.001

To further elucidate how systemic inflammation increased seizure susceptibility, we examined the effect of NOX2 knockout on inflammation in the peripheral and central nervous systems. Peripheral TNFα has been shown as an important mediator of seizure susceptibility following LPS-induced systemic inflammation [4]. Therefore, we first compared TNFα levels in the plasma collected from these WT, gp91^phox−/−^, and p47^phox−/−^ groups of mice 1 h after LPS or vehicle injection. Our data showed that there was no difference in the basal levels of TNFα among the groups and that LPS increased plasma TNFα levels in those three groups with indistinguishable potency (Fig. 2a). We also examined the basal levels of PTZ-seizure susceptibility, the microglial protein marker iba-1, the astrocytic marker glial fibrillary acidic protein (GFAP), and the brain-derived neurotrophic factor (BDNF), a marker related to neuronal excitability [36] in the brains of the wild-type and NOX2-knockout mice without LPS injection. We found no difference in the PTZ-induced seizure susceptibility, including latency to seizure onset and the duration of seizures (stage 4–6) between the WT and NOX2-knockout mice (see Additional file 1). We also found no differences in the expression of these glial and neuronal markers among these groups (see Additional file 1). In addition, they had similar characteristics to those of resting microglia, composed of long branching processes and a small cellular body (see Additional file 1). In response to systemic inflammation, microglia are rapidly activated and undergo morphological changes, consisting of the cell body increasing in size and becoming irregular in shape with thicker and shorter processes (Fig. 2b). Following LPS stimulation, the proportion of activated microglia was significantly lower in the brains (including the hippocampus) of gp91^phox^ and p47^phox^ knockout mice compared with WT mice (Fig. 2b,c). Next, we determined the levels of cytokines that have been shown as contributors to seizure occurrence, including TNFα, IL-1β, CCL2, IL-6, and IL-12, and the anti-inflammatory cytokine IL-10, which may protect against seizure activity. We measured the expression of these cytokines in the brains of the wild-type and NOX2-knockout mice 1.5 h after LPS injection. RT-qPCR analyses showed that these six cytokines were significantly elevated in all genotypes, but the increases of TNFα, IL-1β, IL-6, and CCL2 were significantly attenuated, and the increase of IL-10 was enhanced in NOX2-knockout mice compared with WT mice (Fig. 3). Collectively, these data suggest that NOX2-dependent microglial activation and the ensuing production of proinflammatory cytokines play a key role in the systemic inflammation-elicited increase in seizure susceptibility.

### Primary cultured microglia from NOX2-deleted mice showed reduced proconvulsive factor expression after LPS, TNFα, and IL-1β stimulation

**Fig. 4:**
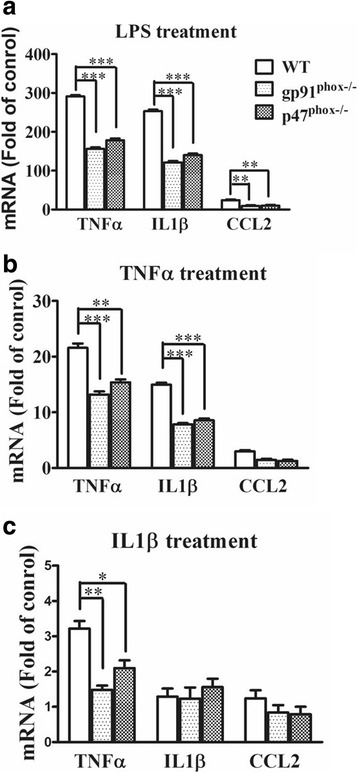
Primary cultured microglia from gp91^phox−/−^ and P47^phox−/−^ mice showed reduced proconvulsive factor expression after LPS, TNFα, and IL1β stimulation. Microglia-enriched cultures from brains of wild-type (WT), gp91^phox−/−^, and P47^phox−/−^mice were seeded (7.5 × 10^4^/well) in 24-well plates and were either treated with LPS 5 ng/ml (**a**), TNFα 500 pg/ml (**b**), or IL-1β 500 pg/ml (**c**) or vehicle for 1 h. Microglia were then subjected to RNA preparation and RT-qPCR analyses. β-actin mRNA expression served as the internal control. The expression of each cytokine of the treated cells was compared to that of the genotype-matched vehicle-treated cultures. Data present the mean ± SEM of three independent experiments performed in triplicate. Bonferroni post hoc test vs. corresponding control; *p < 0.05, **p < 0.01, ***p < 0.001

In this scenario of systemic inflammation and ensuing neuroinflammation, many factors, including LPS and cytokines, may contribute to microglial activation, which in turn produce many proinflammatory factors [37, 38]. To investigate the role of microglial NOX2 in these events, we prepared primary enriched-microglial cultures from the WT and NOX2-knockout mice, and treated these cultures with 5 ng/ml LPS, 500 pg/ml TNFα, or 500 pg/ml IL-1β for 1 h. TNFα and IL-1β are two main important proconvulsive cytokines in the periphery and in the CNS following LPS administration [4, 9, 37]. RT-qPCR analyses showed that LPS treatment significantly enhanced the expression of TNFα, IL-1β, and CCL2 in primary microglia from all three groups of mice, but the induction in NOX2-knockout microglia was approximately 50% lower than that in the WT (Fig. 4a). TNFα significantly enhanced levels of TNFα, IL-1β, and CCL2 transcripts, but the increased levels of TNFα and IL-1β were significantly attenuated in p47^phox−/−^ and gp^91phox−/−^ microglia compared with those of WT microglia (Fig. 4b). Similarly, IL1β stimulation upregulated TNFα mRNA levels in all genotypes, but the levels were significantly lower in p47^phox−/−^ and gp^91phox−/−^ microglia than in WT microglia (Fig. 4c). These results suggested that following LPS (i.p.) stimulation, microglial NOX2 played a critical role in the local production of pro-convulsive cytokines such as TNFα and IL-1β, which in turn enhanced neuronal excitability and seizure susceptibility.

### DPI post-treatment attenuated the increased seizure susceptibility following systemic inflammation

**Fig. 5:**
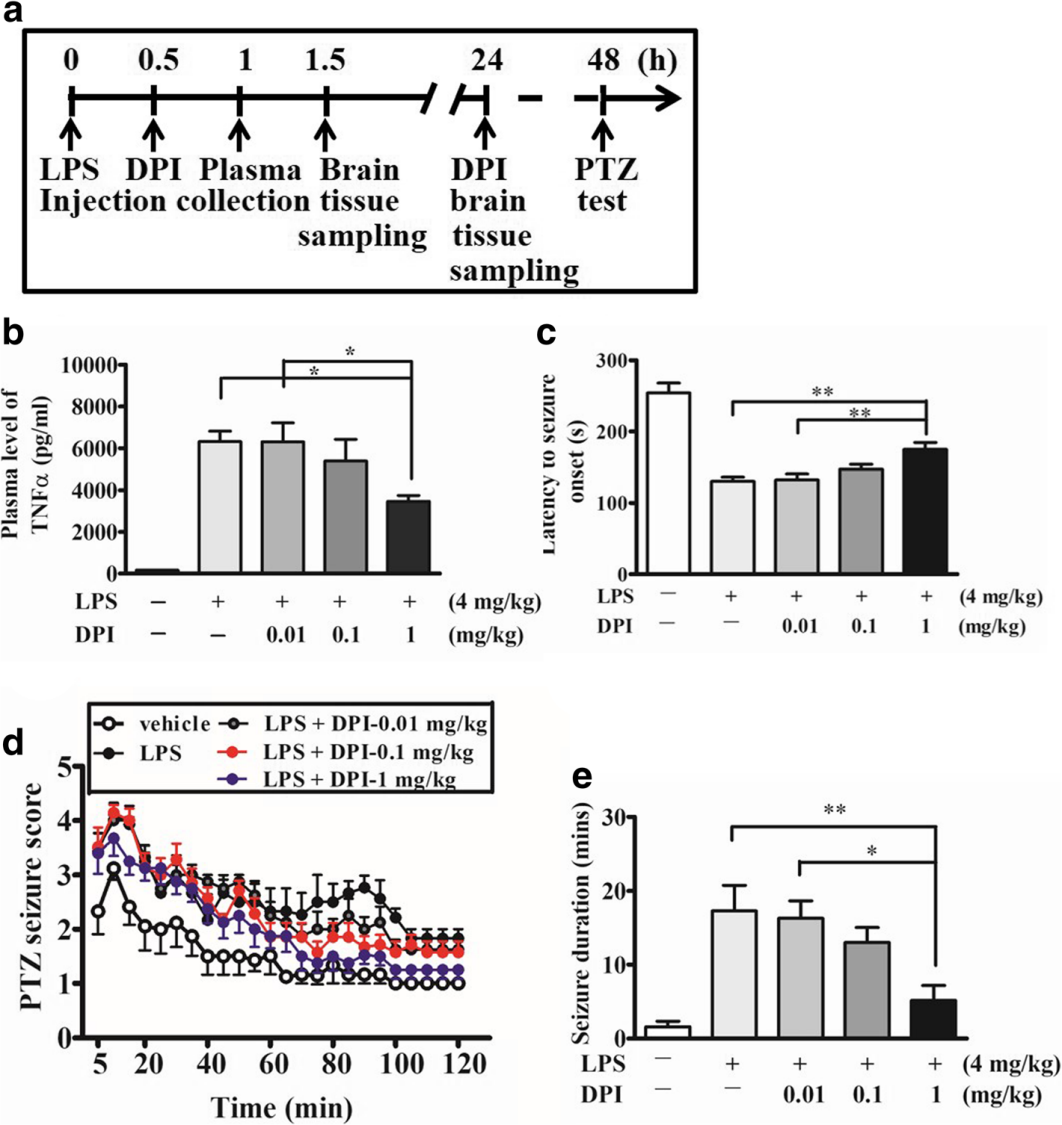
DPI post-treatment attenuated the increased seizure susceptibility following systemic inflammation. **a** The protocol for DPI post-treatment experiments is schematized. Wild-type (WT) mice were either injected intraperitoneally (i.p.) with 4 mg/kg LPS or with vehicle (saline). Then, the mice were treated with 0, 0.01, 0.1, or 1 mg/kg DPI or vehicle (0.025% DMSO) 30 min and 24 h after LPS injection. 48 h later, all the mice received PTZ (60 mg/kg, i.p.) treatment (*n* = 6 or 7 mice/group). **b** Plasma samples were collected 1 h after LPS injection, and the levels of TNFα were determined and compared with that of the vehicle-treated mice. Data present the mean ± SEM; *n* = 5 for each treatment. Bonferroni post hoc test vs. LPS-vehicle-treated group; *p < 0.05. **c** The latency to initial seizure onset (clonic with/without tonic convulsion) after PTZ administration. Data are presented as mean ± SEM. Bonferroni post hoc test vs. LPS-injected vehicle-treated group; **p < 0.01. **d** Seizure susceptibility scored once every 5 min for 2 h after PTZ injection. Two-way repeated measures ANOVA revealed that the main effect for LPS-injectee vehicle-treated and LPS-DPI-treated groups yielded an F ratio of F(3,576) = 18.06, *p* < 0.0001. Bonferroni test analysis further revealed significant difference between the LPS-injected vehicle-treated group LPS-injected 1 mg/kg DPI-treated group (F(1,288) = 41.05; *p* < 0.0001). **e** The total duration (min) of seizure behavior ≥ stage 4. Data present the mean ± SEM. Bonferroni post hoc *t*-test vs. LPS-injected vehicle treatment; *p < 0.05, **p < 0.01

Based on the findings, we propose that NADPH oxidase is a potential pharmacological target to prevent the development of increased seizure susceptibility following systemic inflammation. We therefore investigated the anticonvulsant efficacy of the NADPH oxidase inhibitor DPI in the mouse sepsis model. Wild-type mice were treated with 0.01, 0.1, or 1 mg/kg (i.p.) DPI or vehicle (0.025% DMSO) 30 min and 24 h after LPS administration, and blood samples were collected to measure plasma TNFα 1 h after LPS, and subsequently determined PTZ-induced (60 mg/kg, i.p.) seizure susceptibility (Fig. 5a). As shown in Fig. 5b, 1 mg/kg of DPI significantly decreased plasma TNFα compared with vehicle treatment. The latency to initial seizure onset after PTZ administration was significantly increased in 1 mg/kg DPI-treated WT group, compared with vehicle-treated WT group 48 h after LPS injection (Fig. 5c). In addition, two-way repeated measures ANOVA revealed that the main effect for LPS-injected vehicle-treated and LPS-injected DPI-treated groups yielded an F ratio of F(3,576) = 18.06, *p* < 0.0001, indicating a significant difference between these four groups to PTZ-induced seizure. Further analysis revealed that there is significant difference between the LPS-injected vehicle-treated group and LPS-injected 1 mg/kg DPI-treated group (F(1,288) = 41.05; *p* < 0.0001). The results indicate that 1 mg/kg DPI treatment significantly attenuated the PTZ-induced seizures susceptibility following LPS injection (Fig. 5d). The duration of PTZ-induced seizure (stage 4–6) was also significantly decreased by 1 mg/kg DPI treatment (Bonferroni post hoc test; *p* < 0.01) (Fig. 5e). These results indicated that DPI could attenuate peripheral TNFα levels as well as PTZ-induced seizure susceptibility in LPS-treated mice.

### DPI post-treatment attenuated microglia activation and pro-convulsive cytokine expression in brains after LPS injection

**Fig. 6:**
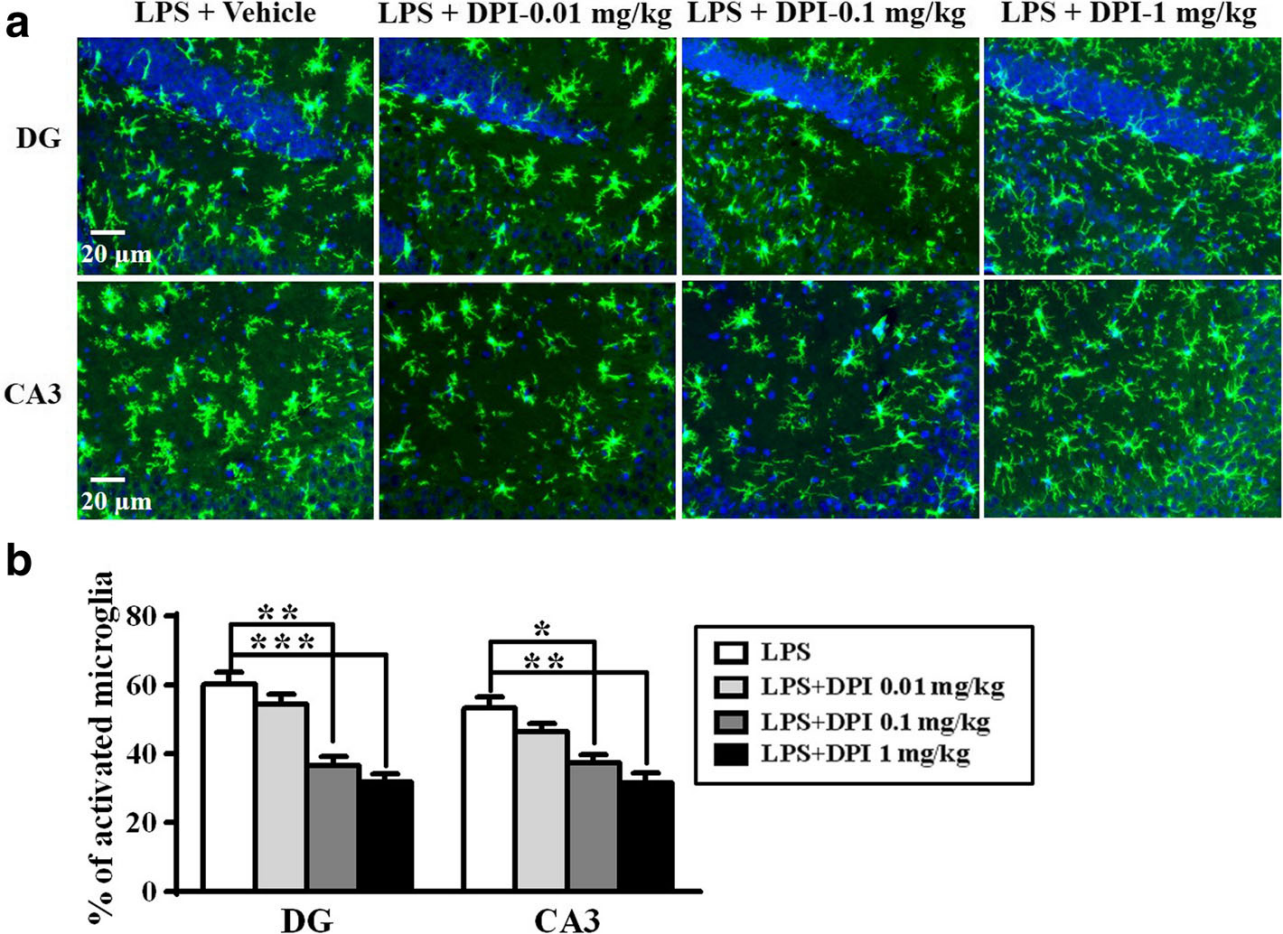
DPI post-treatment attenuated microglia activation in brain after LPS injection. **a** Wild-type mice were treated with a single dose of 0.01, 0.1, or 1 mg/kg DPI or vehicle (0.025% DMSO) 30 min after 4 mg/kg LPS injection (i.p.), and then sacrificed 24 h after LPS. Brain sections were prepared and immunostained for the microglial marker iba-1. Representative images of the stained microglia in the dentate gyrus (DG) and CA3 regions of mouse hippocampus are shown. **b** Activated microglia were identified by increased cell size and irregular shape. The proportions of activated microglia in hippocampus were estimated. Data represent the mean ± SEM of values from three animals per treatment. Bonferroni post hoc test vs. vehicle-treated mouse group; *p < 0.05, **p < 0.01, ***p < 0.001

**Fig. 7:**
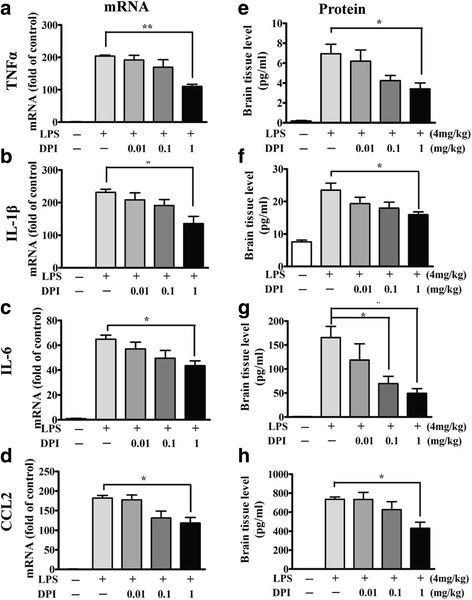
DPI post-treatment attenuated pro-convulsive cytokine expression in brain after LPS injection. Wild-type mice were treated with a single dose of vehicle or DPI at 0.01, 0.1, or 1 mg/kg 30 min after LPS injection. The brains were harvested for assessment of the transcript expression (*n* = 5/group) of several cytokines using RT-qPCR (**a–d**) 1.5 h after LPS injection, and the protein levels (*n* = 3/group) using multiplex assay 24 h after LPS injection (**e–h**), respectively. β-actin expression served as the internal control. The expression of each cytokine of the DPI-treated mice was compared with that of the LPS-injected vehicle-treated control. Data present the mean ± SEM. Bonferroni post hoc test vs. LPS-injected vehicle-treated group; *p < 0.05, **p < 0.01

Subsequent examinations of microglia morphology showed that post-treatment with DPI also decreased the proportion of activated microglia in the mouse CNS following systemic inflammation (Fig. 6a). While LPS induced an approximately 60 and 53% increase of activated microglia in the DG and CA3 regions, respectively, DPI as low as 0.1 mg/kg was able to significantly attenuate microglia activation (Fig. 6b). Post-treatment with 1 mg/kg DPI significantly decreased the upregulation of TNFα, IL-1β, IL-6, and CCL2 mRNA in mouse brain 1.5 h after LPS treatment (Fig. 7a–d). The protein concentrations of these cytokines were measured in brain tissues 24 h after LPS injection using multiplex cytokine assays. DPI at 1 mg/kg, but not at 0.01 or 0.1 mg/kg, significantly attenuated the upregulation of TNFα, IL-1β, IL-6, and CCL2 proteins compared with vehicle treatment (Fig. 7e–h), whereas 0.1 mg/kg DPI was also high enough to decrease IL-6 expression (Fig. 7g). However, there was no difference in the expression of IL-10 and IL-12 between vehicle-treated and DPI-treated mice following LPS injection (see Additional file 2). These results indicated that post-treatment with DPI could inhibit upregulation of proconvulsive cytokines (TNFα, IL-1β, IL-6, and CCL2) at both the mRNA and protein levels in mouse brain following systemic inflammation.

### DPI post-treatment attenuated upregulation of gp91 in brain after LPS stimulation

**Fig. 8:**
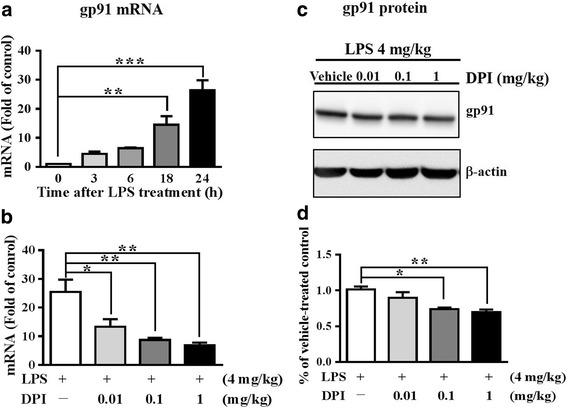
DPI post-treatment attenuated gp91 expression in brain after LPS stimulation in mice. **a** Wild-type mice (*n* = 3/group) were treated with LPS and were sacrificed 0, 3, 6, 18, and 24 h after LPS and the brains were harvested. The transcript levels of gp91 were determined by RT-qPCR analyses and compared with that of the vehicle-treated control (to which a value of 1 was assigned). Data present the mean ± SEM. Bonferroni post hoc test vs. LPS-injected vehicle-treated group; **p < 0.01, ***p < 0.001. **b** The mice were treated with a single dose of vehicle or DPI (0.01, 0.1, or 1 mg/kg) 30 min after LPS injection and then were sacrificed 24 h after LPS. The transcript levels of gp91 were determined by RT-qPCR analyses and compared with that of the LPS-injected vehicle-treated control (to which a value of 1 was assigned). Data present the mean ± SEM. Bonferroni post hoc test vs. LPS-injected vehicle-treated group; *p < 0.05, **p < 0.01. **c** The brains as described in **b** were analyzed by western blot analyses to determine the levels of gp91 protein. Representative blots are shown. **d** The gp91 signals were quantitated and normalized to the internal control. All the normalized values were compared to the LPS-injected vehicle-treated group (to which a value of 1 was assigned). Data represent the mean ± SEM of the results generated from three animals for each group. Bonferroni post hoc test vs. LPS-injected vehicle-treated group; *p < 0.05, **p < 0.01

We further examined whether DPI post-treatment affected NOX expression in mouse brain following LPS injection. RT-qPCR analyses showed that the expression of the gp91 transcript in brain was persistently upregulated for at least 1 day following LPS injection (Fig. 8a). We treated the mice with 0.01, 0.1, or 1 mg/kg DPI 30 min after LPS injection and harvested brains 24 h after LPS injection for RT-qPCR and western blot analyses. The data showed that DPI post-treatment dose-dependently reduced the expression of gp91 transcript (Fig. 8b) and gp91 protein (Fig. 8c,d). In comparison, NOX1 levels were not affected by DPI (see Additional file 3). These results further indicated that DPI post-treatment could also inhibit the deleterious neuroinflammatory response by attenuating the upregulation of NOX2 expression in the mouse brain following LPS stimulation.

## Discussion

In the present study, we demonstrate that the increased seizure susceptibility following LPS-induced systemic inflammation strongly depends on activation of NOX2 in activated microglia. Our findings suggest that NOX2 activation and proinflammatory cytokine generation by microglia sustains the local neuroinflammatory response and increases neuronal excitability, which in turn reduces the threshold for seizure initiation, exacerbating seizure severity. Indeed, mice with NOX2 subunit knockout exhibited reduced microglial activation, attenuated production of the proconvulsive cytokines IL-1β, TNFα, IL-6, and CCL2, and lower seizure susceptibility to PTZ than wild-type mice following LPS-induced inflammation. Similarly, post-treatment with the NADPH oxidase inhibitor DPI following LPS also attenuated microglia activation and the expression of these proconvulsive cytokines in mouse brain. These results strongly suggest that like NOX2 deletion, DPI post-treatment suppresses sepsis-associated neuroinflammation and associated neuronal hyperexcitability, thereby reducing the probability of seizure induction.

The NOX family is comprised of seven members including NOX1-5 and DOUX1-2 [39]. Each isoform is composed of distinct NOX and regulatory subunits but all generate superoxide by transferring electrons across biological membranes. Both NOX1 and NOX2 are co-expressed in microglia but may have distinct functions in microglia activation [40, 41]. In the present study, we clearly demonstrate that NOX2-derived ROS is critical for increasing seizure susceptibility following systemic inflammation via promotion of proconvulsive cytokine expression, although we cannot eliminate a contribution by NOX1. NOX2 is initially found in phagocytic cells (e.g., neutrophils) mediating host defense against microorganisms and also identified in non-phagocytic cells such as astroglia and neurons [42, 43]. NOX-derived ROS levels in non-phagocytic cells are typically much lower than in phagocytic cells since they are not generated to host defense, but as second messengers molecules in response to physiological stimuli (e.g., insulin) [44]. A study using LPS as a stimulus of microglial activation showed that only NOX2 from microglia, and not astrocytes and neurons, caused NADPH oxidase-mediated neuron damage [42]. Another study also showed that immunoreactivity to gp91^phox^ (NOX2 catalytic subunit) was mainly co-localized to activated microglia but co-localized to a few astrocytes and neurons in traumatic brain injury [43]. These studies suggest that even though NADPH oxidase can be activated in cells from the non-myeloid lineage, the resulting production of ROS is significantly lower than in microglia and in innate immunity (e.g., macrophages and neutrophils). Thus, microglia are likely the predominant source of NOX2-derived ROS in brain and thus a more effective target for prevention of neuronal hyperexcitability and seizure induction following sepsis.

Accumulating evidence indicates that systemic inflammation is accompanied by a similar inflammatory response in the CNS resulting from microglial activation and local synthesis of proinflammatory cytokines [4, 6, 15, 45]. Although the precise mechanisms responsible for the initial synthesis of cytokines in the brain are not entirely clear, peripheral TNFα may be a critical trigger by promoting leukocyte-endothelial interactions, microglial activation, and subsequent recruitment of monocytes [46]. We found that greater plasma TNFα concentrations (induced by higher LPS doses, i.e., 4 mg/kg vs. 0.5 mg/kg i.p.) evoked more severe neuroinflammation through enhanced brain accumulation of proinflammatory factors such as TNFα, IL-6, and IL-1β (see Additional file 4). Interestingly, LPS-induced plasma TNFα levels did not differ among gp91^phox−/−^, p47^phox−/−^, and WT mice, either in the current study (Fig. 2a) or in a previous study [47]. However, post-treatment with higher doses of DPI decreased plasma TNFα concentrations in mice following LPS stimulation. One possibility is that DPI, which is a general flavoprotein inhibitor, blocks other NOX isoforms as well as xanthine oxidase [48, 49]. Although it remains unclear whether genetic deletion of NOX2 subunits and DPI administration have distinct effects on other NOX enzymes (e.g., through compensatory changes in NOX isoform expression and non-selective pharmacological inhibition), our results suggest that DPI has additional protective benefits against neuroinflammation by inhibiting the production of peripheral inflammatory factors (e.g., TNFα).

There is compelling evidence that proinflammatory cytokines can induce neuronal hyperexcitability and thus contribute to the development of epilepsy [7, 15]. It is believed that these effects are mediated by direct and indirect upregulation of excitatory glutamatergic transmission and downregulation of inhibitory GABAergic transmission [50, 51]. NOX2 is one of the major sources of ROS for cellular signaling. The present study demonstrates that both genetic deletion of NOX2 subunits and DPI post-treatment can strongly inhibit proconvulsive cytokine gene expression in response to systemic inflammation (Figs. 3 and 7) and that microglia are a major source of these cytokines (Fig. 4). In addition, DPI attenuated NOX2 upregulation following sepsis (Fig. 8b, c), but had no effect on NOX1 expression (Additional file 3). These results support the hypothesis that DPI attenuates neuronal hyperexcitability by inhibiting NOX2 overexpression and NOX2-derived ROS signaling pathways linked to proconvulsive cytokine release.

Given the potential role of proconvulsive cytokines in mediating neuronal hyperexcitability following systemic inflammation, targeting these factors is considered a potential therapeutic strategy for suppressing the development of post-sepsis epilepsy [7, 52]. Moreover, targeting the early stages of neuroinflammation following systemic inflammation may prevent both inflammation- and seizure-associated brain damage due to oxidative stress and excitotoxicity. Based on the underlying molecular mechanism of these proconvulsive cytokines and their signaling pathways, several new immunotherapeutic approaches such as IL-1β-converting enzyme inhibitors and suppression of NF-kB are currently in the experimental phase of development for drug-resistant epilepsy [52–54]. However, it still has not been established whether a single proconvulsive cytokine inhibitor such as a TNFα blocker is sufficient to prevent neuroinflammation-mediated neuronal hyperexcitability. In fact, following sepsis, multiple cytotoxic factors such as cytokines/chemokines, ROS, and NOS are released by activated microglia and can both damage neurons directly and induce hyperexcitability. Consequently, a self-sustaining cycle is created through interactions between damaged neurons and dysregulated microglia, which may eventually result in chronic neuroinflammation, epilepsy development, and progressive neurodegeneration [6, 7, 55]. NOX2 complexes that drive ROS signaling pathways are essential mediators of this process [24]. It is thus reasonable to speculate that inhibiting NADPH oxidases can reduce the accumulation of neurotoxic factors (e.g., cytokines, and ROS) and disrupt the development of this vicious pathogenic cycle [24, 55]. Our findings indicated that NOX2 subunit deletion and pharmacological NOX2 inhibition reduced the upregulation of a broad spectrum of proconvulsive cytokines following LPS injection and attenuated subsequent increased seizure susceptibility.

A few studies have examined the therapeutic potential of preventing neuronal hyperexcitability associated with neuroinflammation. Hernandes and colleagues reported that pretreatment with the NOX2 inhibitor apocynin was effective in preventing the development of long-term cognitive impairment from sepsis-associated neuroinflammation [56]. Suppression of COX2-mediated inflammation is also a potential strategy for reducing neuronal hyperexcitability since COX-2 mRNA expression and PGE2 production are widely induced in rodent brain following sepsis [57]. In fact, a COX-2 inhibitor ameliorated the increase in KA-induced seizure susceptibility following sepsis via inhibition of NOX2 signaling [57, 58]. Wang and colleagues reported that post-treatment with ultra-low doses of DPI (10 ng/kg/day) for 2 weeks prevented dopaminergic neurodegeneration and motor deficits in Parkinson’s disease models through inhibition of chronic neuroinflammation [59]. In the present study, a single dose of 0.01 mg/kg DPI significantly reduced the expression of proconvulsive cytokine mRNA and gp91^phox^ mRNA within 24 h after LPS injection, but only higher doses (1 mg/kg) attenuated seizure susceptibility. Collectively, these findings suggest that pharmacological NOX2 inhibitors may suppress the sepsis-associated increase in seizure susceptibility and resulting neurodegeneration by preventing microglial activation and downstream neuroinflammatory signaling.

## Conclusions

In conclusion, this study shows that NOX2 contributes to neuronal hyperexcitability and increased seizure susceptibility in the LPS animal model of sepsis. Our data strongly suggest that pharmacological inhibition of NOX2-derived ROS may be an effective strategy to prevent sepsis-associated neuroinflammation, neuronal hyperexcitability, and seizures.

## Additional file

Additional file 1: Basal seizure susceptibility, molecular levels of cell populations, and morphology of microglia in wild-type and NADPH-oxidase knockout mice. (A) The latency to initial seizure onset (clonic with/without tonic convulsion) after PTZ administration. Data are presented as mean ± SEM. Bonferroni post hoc test vs. LPS-treated WT group; *p* > 0.05. (B) Basal seizure susceptibility scored once every 5 min over the 2-h period to 60 mg/kg PTZ (i.p.) in wild-type (WT), gp91^phox−/−^, and P47 ^phox−/−^ mice (*n* = 6 or 7 per genotype). (C) The total duration (min) of seizure behavior ≥ stage 4 in gp91^phox−/−^, P47 ^phox−/−^, and WT mice. Data represent the mean ± SEM. Bonferroni post hoc test vs. WT mouse group. (D) Representative immunoblots showing the basal protein levels of Iba-1 (microglia marker), GFAP (astroglia marker), and BDNF (neuronal marker) in brains of WT, gp91^phox−/−^ and P47 ^phox−/−^ mice (*n* = 3 per genotype). Bonferroni post hoc test vs. WT mouse group. (E) Photomicrograph of iba1-staining microglia from the dentate gyrus regions (DG) and CA3 regions of each genotype mice (n = 3 per genotype). Resting microglia are composed of a small cellular body and long branching processes (arrow). (TIF 15422 kb)

Additional file 2: DPI post-treatment had no effect to attenuate IL10on attenuating IL-10 and IL-12 expression in brain after LPS injection. Wild-type mice were treated with a single dose of vehicle or DPI at 0.01, 0.1, or 1 mg/kg 30 min after LPS injection. At 1.5 and 24 h after LPS injection, mice were sacrificed and brain extracts prepared for analysis of cytokine transcript (*n* = 5/group) and protein expression (*n* = 3/group) by real-time PCR and multiplex assay, respectively. The transcript levels of IL-10 (A), and IL-12 (B) at 1.5 h after LPS injection. Data are presented as mean ± SEM. Bonferroni post hoc test vs. LPS-injected vehicle-treated group, *p* > 0.05. The protein levels of IL-10 (C), and IL-12 (D) in brain at 24 h after LPS injection. Values are presented as mean ± SEM. Bonferroni post hoc test vs. LPS-injected vehicle-treated control. (TIF 13787 kb)

Additional file 3: DPI post-treatment after LPS stimulation had no significant effect on NOX1 expression. C57BL/6 mice were treated with a single dose of vehicle or DPI 30 min after LPS injection (4 mg/kg; i.p.) and sacrificed 24 h after LPS injection. Representative immunoblots showing the levels of NADPH oxidase 1 (NOX1) protein in vehicle- or DPI-treated mouse brain are shown (A). Quantitative analysis indicated that NOX1 protein expression was not significantly changed by DPI post-treatment (B). Data represent the mean ± SEM of three animals per treatment group. Bonferroni post hoc test vs. LPS-injected vehicle-treated group; *p* > 0.05. (TIF 8723 kb)

Additional file 4: Dose response of LPS on plasma TNFα levels and cytokine gene expression in mouse brain. Male C57BL/6J mice were treated with a lower dose (0.5 mg/kg) or a higher dose (4 mg/kg) of LPS. (A) Plasma levels of TNF-α 1 h after LPS stimulation. Student’s *t*-test; n = 5/group; ***p < 0.001. (B) The levels of TNFα, IL-1β, and IL-6 transcripts in brain 1.5 h after LPS stimulation (n = 3/group). The mRNA levels were calculated relative to expression in matched saline-treated mice. β-actin mRNA was the internal control. Data present the mean ± SEM. Student’s t-test; ***p < 0.001. (TIF 10736 kb)

## Abbreviations

BDNF: Brain-derived neurotrophic factor
CCL2: C-C motif chemokine ligand 2
DPI: Diphenyleneiodonium
GFAP: Glial fibrillary acidic protein
Iba-1: Ionized calcium binding adaptor molecule 1
IL-12: Interleukin 12
IL-1β: Interleukin 1β
IL-6: Interleukin 6
LPS: Lipopolysaccharide
NOX2: NADPH oxidase 2
PTZ: Pentylenetetrazol
ROS: Reactive oxygen species
TNFα: Tumor necrosis factor-alpha
