# Supplementary figures and images for: NADPH oxidases as potential pharmacological targets against increased seizure susceptibility after systemic inflammation
Source: J Neuroinflammation. 2018 May 12;15:140. doi: 10.1186/s12974-018-1186-5 (PMC5948699; doi:10.1186/s12974-018-1186-5)

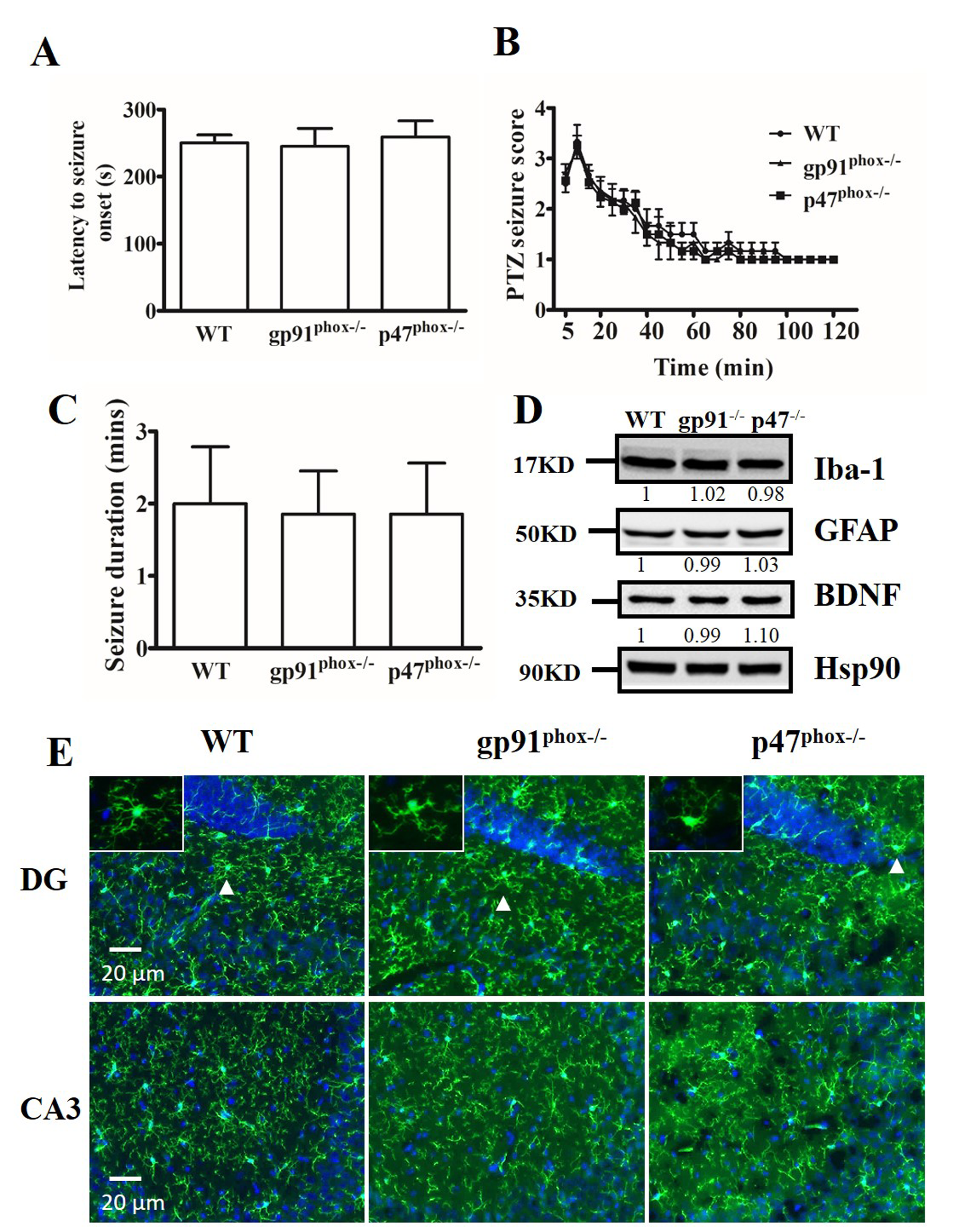

Supplement: Supplementary file 1 — Basal seizure susceptibility, molecular levels of cell populations, and morphology of microglia in wild-type and NADPH-oxidase knockout mice. (A) The latency to initial seizure onset (clonic with/without tonic convulsion) after PTZ administration. Data are presented as mean ± SEM. Bonferroni post hoc test vs. LPS-treated WT group; p > 0.05. (B) Basal seizure susceptibility scored once every 5 min over the 2-h period to 60 mg/kg PTZ (i.p.) in wild-type (WT), gp91phox−/−, and P47 phox−/− mice (n = 6 or 7 per genotype). (C) The total duration (min) of seizure behavior ≥ stage 4 in gp91phox−/−, P47 phox−/−, and WT mice. Data represent the mean ± SEM. Bonferroni post hoc test vs. WT mouse group. (D) Representative immunoblots showing the basal protein levels of Iba-1 (microglia marker), GFAP (astroglia marker), and BDNF (neuronal marker) in brains of WT, gp91phox−/− and P47 phox−/− mice (n = 3 per genotype). Bonferroni post hoc test vs. WT mouse group. (E) Photomicrograph of iba1-staining microglia from the dentate gyrus regions (DG) and CA3 regions of each genotype mice (n = 3 per genotype). Resting microglia are composed of a small cellular body and long branching processes (arrow). (TIF 15422 kb) [file 12974_2018_1186_MOESM1_ESM.tif]

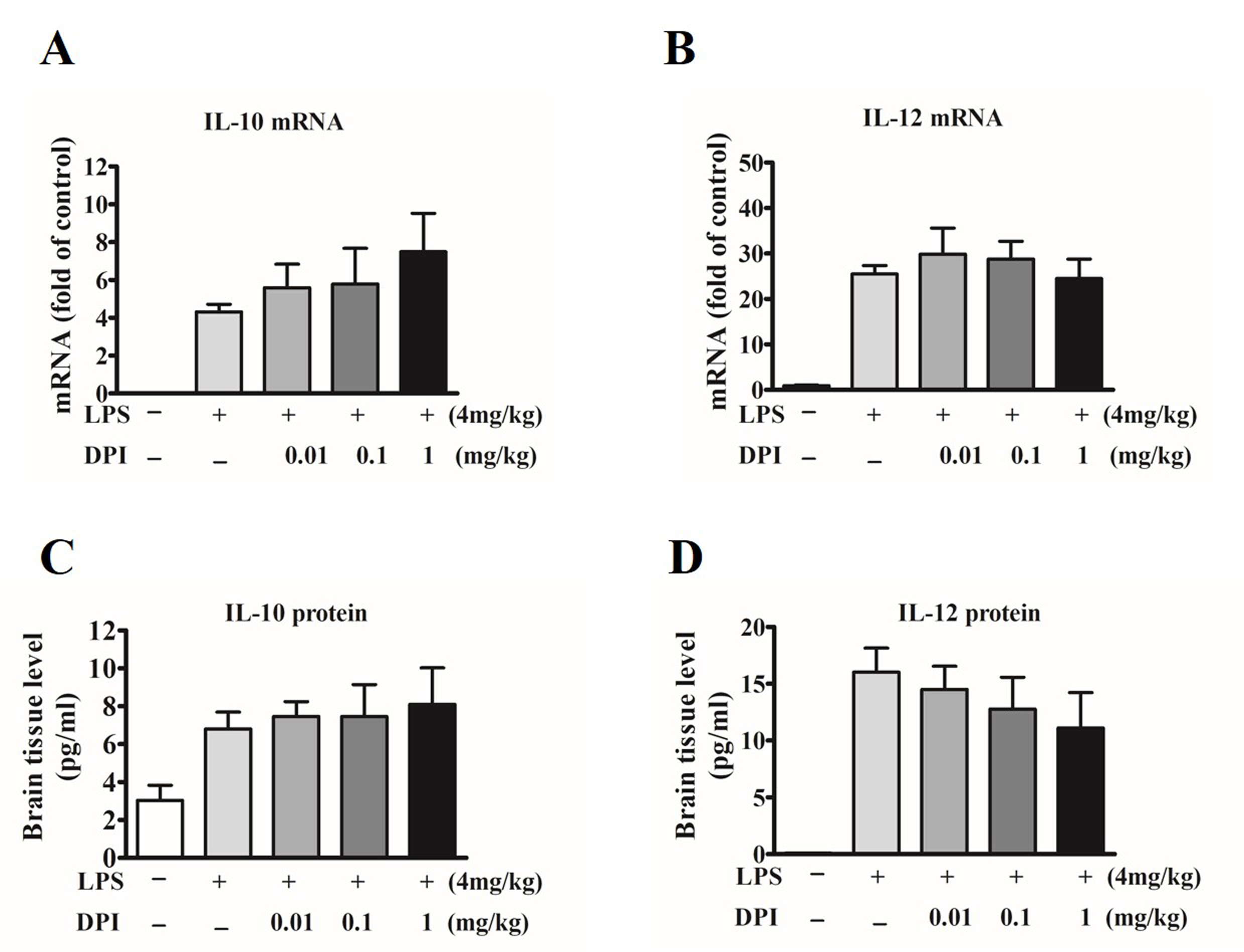

Supplement: Supplementary file 2 — DPI post-treatment had no effect to attenuate IL10on attenuating IL-10 and IL-12 expression in brain after LPS injection. Wild-type mice were treated with a single dose of vehicle or DPI at 0.01, 0.1, or 1 mg/kg 30 min after LPS injection. At 1.5 and 24 h after LPS injection, mice were sacrificed and brain extracts prepared for analysis of cytokine transcript (n = 5/group) and protein expression (n = 3/group) by real-time PCR and multiplex assay, respectively. The transcript levels of IL-10 (A), and IL-12 (B) at 1.5 h after LPS injection. Data are presented as mean ± SEM. Bonferroni post hoc test vs. LPS-injected vehicle-treated group, p > 0.05. The protein levels of IL-10 (C), and IL-12 (D) in brain at 24 h after LPS injection. Values are presented as mean ± SEM. Bonferroni post hoc test vs. LPS-injected vehicle-treated control. (TIF 13787 kb) [file 12974_2018_1186_MOESM2_ESM.tif]

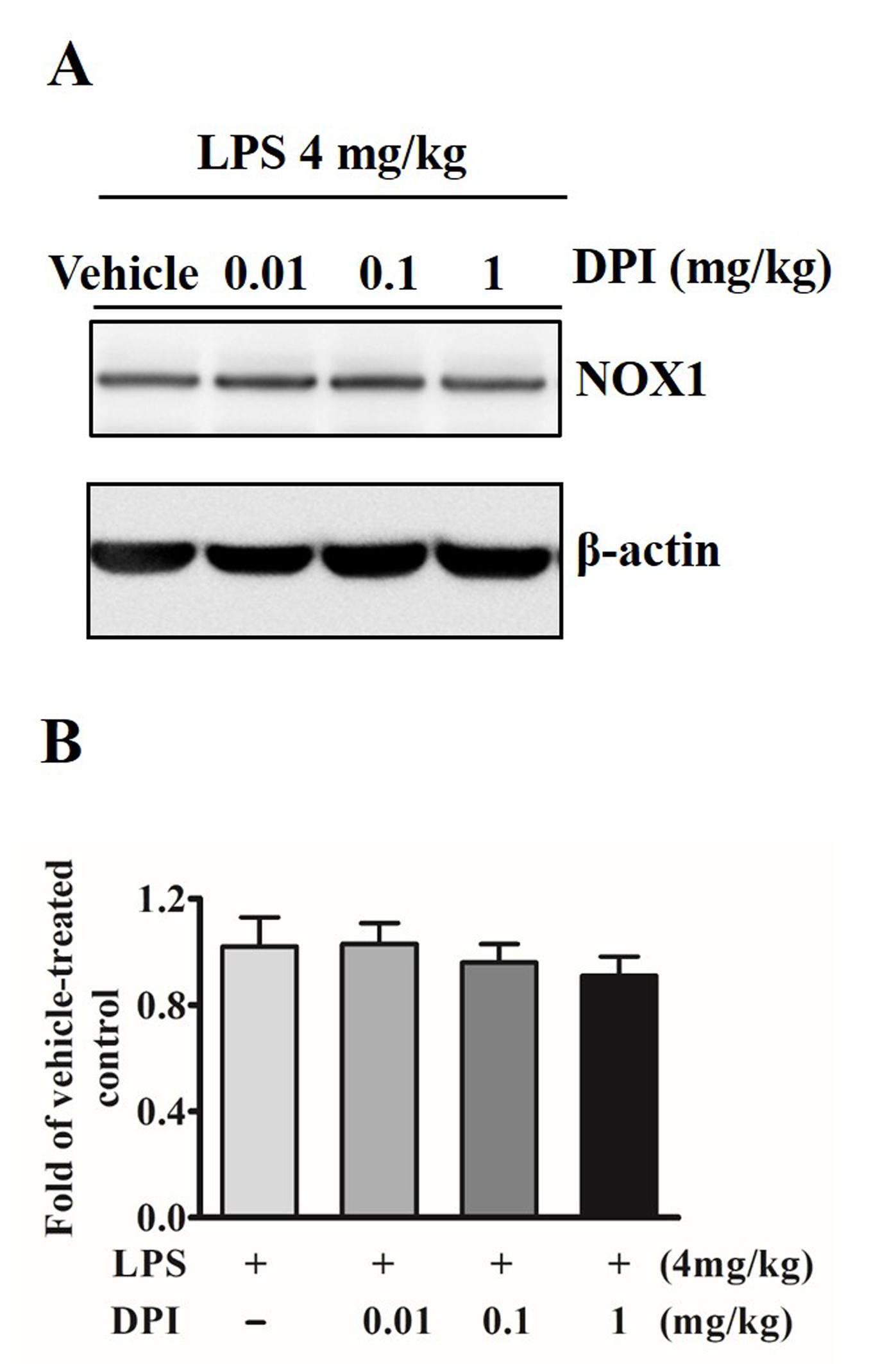

Supplement: Supplementary file 3 — DPI post-treatment after LPS stimulation had no significant effect on NOX1 expression. C57BL/6 mice were treated with a single dose of vehicle or DPI 30 min after LPS injection (4 mg/kg; i.p.) and sacrificed 24 h after LPS injection. Representative immunoblots showing the levels of NADPH oxidase 1 (NOX1) protein in vehicle- or DPI-treated mouse brain are shown (A). Quantitative analysis indicated that NOX1 protein expression was not significantly changed by DPI post-treatment (B). Data represent the mean ± SEM of three animals per treatment group. Bonferroni post hoc test vs. LPS-injected vehicle-treated group; p > 0.05. (TIF 8723 kb) [file 12974_2018_1186_MOESM3_ESM.tif]

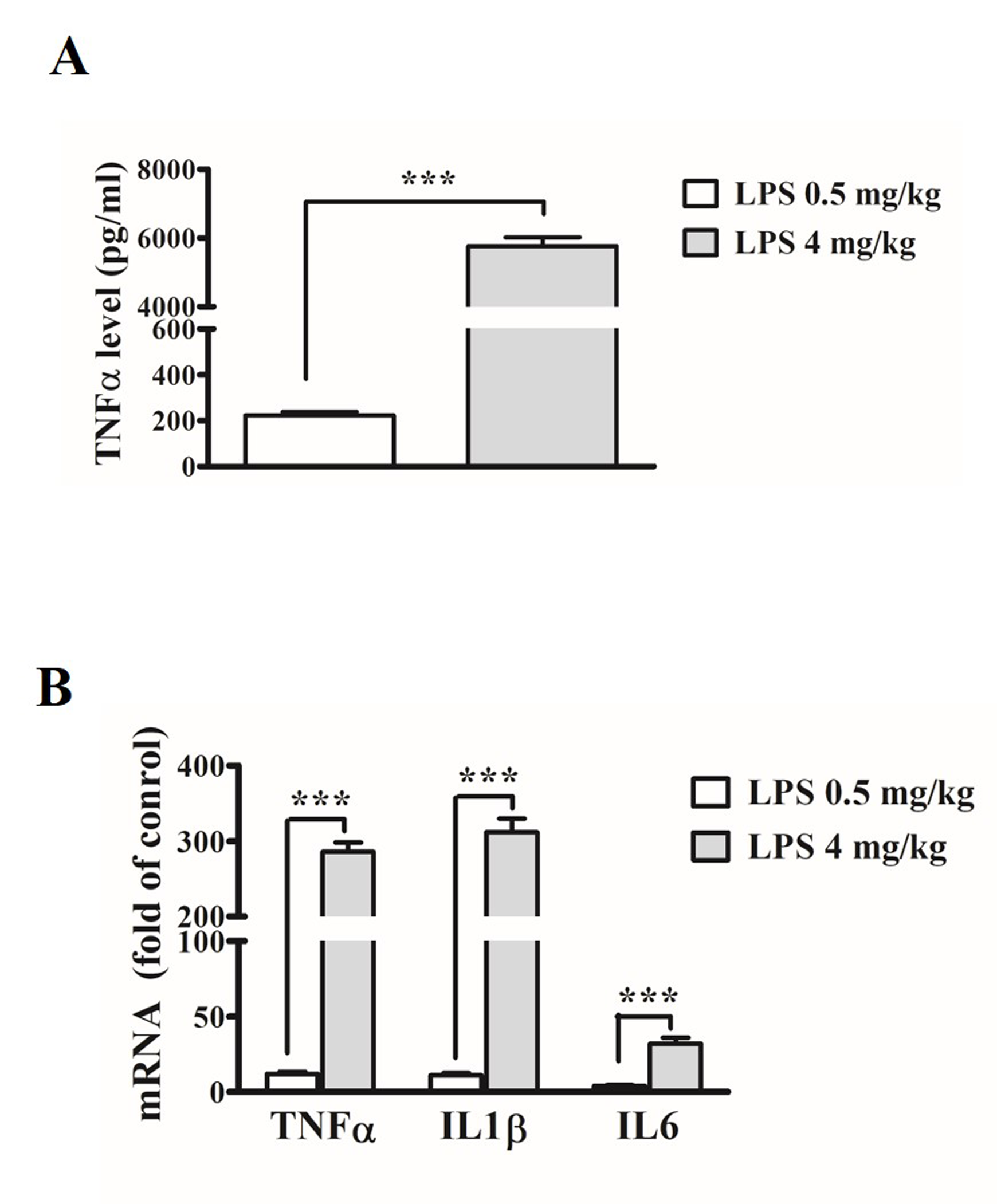

Supplement: Supplementary file 4 — Dose response of LPS on plasma TNFα levels and cytokine gene expression in mouse brain. Male C57BL/6J mice were treated with a lower dose (0.5 mg/kg) or a higher dose (4 mg/kg) of LPS. (A) Plasma levels of TNF-α 1 h after LPS stimulation. Student’s t-test; n = 5/group; ***p < 0.001. (B) The levels of TNFα, IL-1β, and IL-6 transcripts in brain 1.5 h after LPS stimulation (n = 3/group). The mRNA levels were calculated relative to expression in matched saline-treated mice. β-actin mRNA was the internal control. Data present the mean ± SEM. Student’s t-test; ***p < 0.001. (TIF 10736 kb) [file 12974_2018_1186_MOESM4_ESM.tif]
